# Kaolin-Gallic Acid Functionalized Gamma-Chitosan/Alginate Beads: A Novel Material for Chlorhexidine and Cetylpyridinium Elimination from Dental Effluents

**DOI:** 10.1021/acsomega.5c12001

**Published:** 2026-03-02

**Authors:** Titiya Meechai, Prach Ponlawat, Hattapol Kumchai, Jintapat Nateewattana, Woravith Chansuvarn, Tanutta Amnuaywattanakul, Anchana Kuttiyawong, Benjapat Wongpaibool, Thongnard Kumchai, Phitchan Sricharoen

**Affiliations:** † Faculty of Dentistry, 232369 Bangkokthonburi University, Thawi Watthana, Bangkok 10170, Thailand; ‡ Division of Health, Cosmetic and Anti-Aging Technology, Faculty of Science and Technology, 187381 Rajamangala University of Technology Phra Nakhon, Bangkok 10800, Thailand; § Faculty of Science and Technology, 187381 Rajamangala University of Technology Phra Nakhon, Bangkok 10800, Thailand

## Abstract

Dental effluents often contain persistent cationic antiseptics, such as chlorhexidine gluconate (CHG) and cetylpyridinium chloride (CPC). These substances raise environmental concerns due to their widespread clinical use, antimicrobial persistence, and poor biodegradability. This study investigates the synthesis and evaluation of kaolin-gallic acid functionalized gamma-chitosan/alginate (γCS-AG/KA-GA) composite beads for the adsorption of CHG and CPC from aqueous solutions and real dental wastewater. The composite beads were prepared by ionic cross-linking of gamma-irradiated chitosan and alginate in a CaCl_2_ bath. Kaolin and gallic acid were incorporated to enhance both structural stability and adsorption functionality. Comprehensive characterization techniques, including high-resolution transmission electron microscopy (HRTEM), Brunauer–Emmett–Teller (BET) analysis, Fourier-transform infrared spectroscopy (FTIR), Energy-dispersive X-ray spectroscopy (EDS), and X-ray diffraction (XRD), confirmed the successful formation of a porous hybrid composite with an increased surface area of 68.9 m^2^·g^–1^. Batch adsorption experiments showed rapid uptake of both antiseptics within 60 min, reaching equilibrium at 180 min. The adsorption behavior was well described by the Langmuir isotherm (*R* ^2^ > 0.98) and the pseudo-second-order kinetic model (*R* ^2^ ≈ 0.99), resulting in maximum adsorption capacities of 82.7 mg·g^–1^ for CHG and 75.3 mg·g^–1^ for CPC. When applied to authentic dental wastewater collected from the BTU Dental Clinic, the γCS-AG/KA-GA beads achieved a removal efficiency of 65 ± 3%. This slight reduction in performance compared to synthetic systems was attributed to ionic competition and matrix fouling. Overall, these results demonstrate that γCS-AG/KA-GA composite beads provide a promising, sustainable, and material-based approach to reducing antiseptic contamination in dental effluents and promoting greener dental wastewater management strategies.

## Graphical abstract

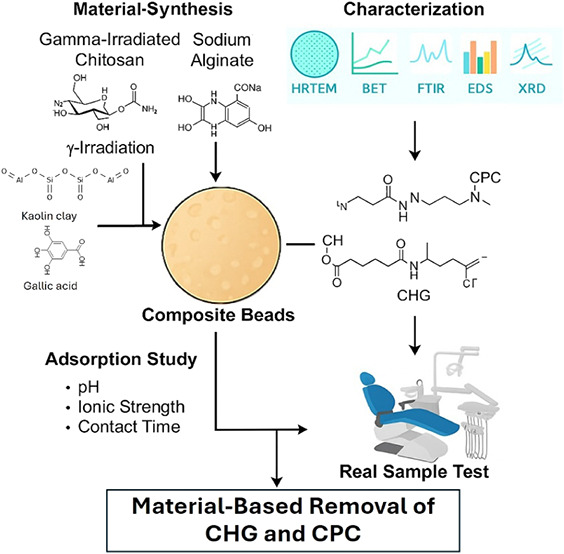

## 1 Introduction

Dental wastewater is a complex and increasingly important environmental issue arising from routine dental care practices. This wastewater typically contains mercury residues from amalgam fillings, trace metals from dental instruments, organic matter from saliva and blood, and various oral antiseptics commonly used in dental treatments. Among these substances, two compounds of particular environmental concern are chlorhexidine gluconate (CHG) and cetylpyridinium chloride (CPC). CHG, which is used at concentrations of 0.12–0.2%, is widely considered the gold standard for antiseptic treatment in periodontal therapy and postsurgical oral care. On the other hand, CPC, at concentrations of 0.05–0.07%, is a common active ingredient in many commercial mouthwashes, including brands like Colgate Plax and Crest Pro-Health. While both CHG and CPC are clinically effective, their continuous discharge into wastewater systems raises significant concerns due to their high persistence, low biodegradability, and potential ecological impacts. Studies have shown that CHG maintains strong antimicrobial activity, whereas CPC, a quaternary ammonium surfactant, readily adsorbs onto organic and inorganic particles. This could potentially alter microbial communities and contribute to the development of antimicrobial resistance (AMR).^1–4^

The synthesis of adsorbent materials for contaminant removal is considered an effective and promising technology, offering high efficiency, simplicity, and suitability for practical applications.^5^ Conventional treatment methods for dental and healthcare wastewater, such as coagulation-flocculation, activated carbon adsorption, and membrane-based filtration, have been widely used.^6^ However, these techniques often have significant limitations, including high operational costs, membrane fouling, the generation of secondary waste, and limited effectiveness against a diverse range of pharmaceutical contaminants. As a result, there has been increasing interest in adsorption-based treatments that use biodegradable,^7,8^ environmentally friendly materials.^9^ In this regard, natural biopolymers such as chitosan and alginate have emerged as promising options due to their abundance, low toxicity, and functional groups (−NH_2_, −OH, and −COO^–^). These functional groups can effectively interact with cationic pollutants through electrostatic attraction and hydrogen bonding.^10,11^ To enhance adsorption performance, researchers have actively explored both chemical and physical modifications of biopolymers.

One effective strategy for modifying chitosan is gamma irradiation, which reduces molecular weight, improves solubility, and creates additional reactive functional sites.^12–14^ These structural modifications significantly enhance adsorption efficiency by increasing the accessible surface area and strengthening interactions with target molecules. Previous studies have shown that gamma-irradiated chitosan, when combined with alginate, demonstrates superior mechanical stability and adsorption performance compared to unmodified chitosan systems. Chitosan-alginate composite beads have been successfully used for the removal of dyes and heavy metals from aqueous solutions. However, research specifically focused on the adsorption and removal of dental antiseptics like CHG and CPC remains limited, particularly under conditions relevant to actual dental wastewater. Additionally, inorganic and phenolic reinforcements have been explored as effective methods to further improve the structural integrity and functionality of biopolymer-based adsorbents. Kaolin clay, a naturally occurring aluminosilicate, has been reported to enhance mechanical strength while also introducing negatively charged surface sites that promote the adsorption of cationic species.^15–20^ Gallic acid (GA), a naturally occurring polyphenol, has garnered attention as a multifunctional additive and is widely distributed in plants, including cereals such as rice.^21^ It can serve as a cross-linking agent while also offering aromatic and hydroxyl functional groups. Gallic acid was selected as a functionalizing agent due to its high density of phenolic hydroxyl groups and aromatic structure, which provide stronger hydrogen-bonding and π–π interactions compared to mono- or diphenolic compounds. In addition, its low molecular weight and good water compatibility make it suitable for incorporation into biopolymer matrices without blocking active adsorption sites.^22^ These properties enhance hydrogen bonding and π–π interactions with aromatic cationic compounds, such as CHG and CPC. In our previous research, we successfully developed alginate-gamma-irradiated chitosan composite beads for the adsorption of organic dyes.

This work demonstrated that gamma irradiation significantly enhanced the surface area, pore distribution, and adsorption efficiency of the beads. Building on this foundation, the present study aims to extend the application of gamma-irradiated chitosan/alginate systems for the remediation of dental wastewater by incorporating kaolin and gallic acid as synergistic reinforcing components. The novelty of this research lies in the integrated design of a hybrid biopolymer composite that combines (i) the enhanced reactivity of gamma-irradiated chitosan, (ii) the structural reinforcement and negative charge contribution of kaolin, and (iii) the multifunctional binding capability of gallic acid. The specific objectives of this study include synthesizing kaolin-gallic acid functionalized gamma-chitosan/alginate (γCS-AG/KA-GA) composite beads and systematically characterizing their structural and physicochemical properties using high-resolution transmission electron microscopy (HRTEM), Brunauer–Emmett–Teller (BET) analysis, Fourier-transform infrared spectroscopy (FTIR), Energy-dispersive X-ray spectroscopy (EDS), and X-ray diffraction (XRD). Additionally, we will evaluate the adsorption performance of the composite beads toward CHG and CPC under various conditions, including pH, ionic strength, contact time, and initial concentration. Finally, the applicability of the developed adsorbent will be assessed using real dental wastewater collected from a clinical setting. The outcomes of this study are expected to provide valuable insights into developing sustainable, effective material-based strategies to mitigate antiseptic contamination in dental wastewater systems.

## 2 Materials and Methods

### 2.1 Materials

Chitosan was irradiated with γ rays at a sterilizing dose of 40 kGy. This process resulted in a molecular weight of 190 kDa and a degree of deacetylation of 95%. The chitosan was supplied by the Thailand Institute of Nuclear Technology (Public Organization). Sodium alginate, the primary natural polymer used, was also sourced from Biolife (Thailand). Kaolin clay (analytical grade) was purchased from Merck (Germany), and gallic acid (99%) was obtained from Sigma-Aldrich (USA) as a reinforcing additive. Calcium chloride (99%), also from Sigma-Aldrich (USA), served as the cross-linking agent. For the model dental antiseptics, chlorhexidine gluconate (CHG, 20% solution) and cetylpyridinium chloride (CPC, 98%) were acquired from Sigma-Aldrich and TCI Chemicals (Japan), respectively. Additionally, acetic acid (1% v/v, TCI Chemicals, Japan) and sodium hydroxide (NaOH, 98%, Merck, Germany) were included in the procedures. All solutions were prepared using deionized water.

### 2.2 Preparation of Composite Beads (γCS-AG/KA-GA)

Sodium alginate was initially dissolved in deionized water at 1% (w/v). The mixture was then continuously stirred at low temperatures to ensure uniform dissolution. Kaolin clay, at a concentration of 0.3 g per 100 mL, was predispersed using ultrasonication and then gradually added to the alginate solution. A solution of gallic acid (0.1% w/v) was prepared in deionized water, adjusted to pH 5.5–6.0, and added dropwise to the alginate-kaolin mixture, which was then stirred to achieve homogeneity. In parallel, gamma-irradiated chitosan was dissolved in 1% acetic acid at 2% (w/v) to produce a clear, viscous solution. This chitosan solution was combined with a calcium chloride solution (2% w/v) to create the gelling bath. The alginate-kaolin-gallic acid mixture was loaded into a syringe fitted with a 22G needle and carefully dropped into the calcium chloride-chitosan bath while stirring gently. The ionic cross-linking process led to the instantaneous formation of spherical beads. The beads were allowed to harden for 1 h, then collected and thoroughly rinsed with deionized water until the pH was neutral. The resulting beads were dried using one of two methods: (i) air-drying at room temperature overnight, followed by oven drying at 50 °C for 2 h, or (ii) freeze-drying to preserve the porous structure. The dried beads, designated γCS-AG/KA-GA, were stored in a desiccator until further analysis and adsorption experiments.

### 2.3 Characterization of Composite Beads

The crystalline structure of the prepared composites was analyzed using X-ray diffraction (XRD) with a Bruker D8 Advance A25 diffractometer, equipped with a nickel filter (Cu Kα radiation, λ = 0.154184 nm) and a Lynxeye multistrip detector. High-resolution transmission electron microscopy (HRTEM) images were obtained using a JEOL JEM-ARM200F microscope, equipped with a high-angle annular dark-field (HAADF) detector and an energy-dispersive spectrometer (EDS) for elemental analysis. The specific surface area and porosity of the composites were determined using nitrogen (N_2_) adsorption–desorption isotherms at 77.3 K on a Quantachrome Instruments system, version 11.0, applying the Brunauer–Emmett–Teller (BET) method. Fourier-transform infrared (FTIR) spectra were recorded with a Bruker ALPHA spectrometer (Hong Kong Ltd.). The absorbance of CHG and CPC solutions was measured using a DLAB SP-UV1000 Spectrophotometer.

### 2.4 Adsorption Experiments

Batch adsorption experiments were conducted using aqueous solutions of CHG and CPC at environmentally relevant concentrations. Unless otherwise stated, an initial concentration of 1 mg·L^–1^ was employed to simulate realistic dental wastewater conditions and to maintain the structural integrity of the biopolymer-based composite beads under strongly cationic conditions.

The effects of solution pH (3–9), ionic strength (0–100 mM NaCl), and contact time (0–300 min) were systematically investigated under controlled batch conditions. In each experiment, 0.2–0.5 g of γCS-AG/KA-GA composite beads was added to 25 mL of antiseptic solution, and the mixture was agitated at 250 rpm at 25 ± 1 °C. Preliminary screening experiments indicated that adsorption equilibrium was achieved within 180 min; therefore, this contact time was selected for subsequent adsorption studies.

At predetermined time intervals, aliquots were withdrawn, filtered, and analyzed using UV–vis spectrophotometry at wavelengths of 255–260 nm to determine the residual concentrations of CHG and CPC. Calibration procedures and analytical performance of the UV–vis method were evaluated to ensure reliable quantification. The adsorption capacity (*q*_*e*_, mg·g^–1^) and removal efficiency (%) were calculated using standard mass balance equations. The adsorption capacity (*q*_*e*_, mg·g^–1^) and removal efficiency (%) were calculated using standard equations.^5,23^

$$q_{e}=\frac{{(C}_{0\;}{-\;C}_{e})\times V}{m}$$

$$\mathrm{Removal}\;\mathrm{efficiency}\;\left(\%\right)=\frac{{(C}_{0}{-C}_{e})}{C_{0}}\times 100$$

Where *C*_0_ and *C_e_* (mg/L) are the initial and equilibrium concentrations of CHG or CPC, respectively, *V* is the solution volume (L), and *m* is the adsorbent mass (g).

### 2.5 Real Sample Test

To evaluate the effectiveness of the composite beads, wastewater samples were collected directly from the dental chair drainage at the BTU Dental Clinic, Bangkokthonburi University, Thailand, after routine treatments involving CHG and CPC. The samples were filtered through a 0.45 μm membrane to remove solids and then stored at 4 °C. Key physicochemical parameters, including pH, conductivity, total dissolved solids (TDS), and chemical oxygen demand (COD), were measured according to the APHA Standard Methods (2017). Batch adsorption experiments were conducted, and concentrations of CHG and CPC were quantified using UV–vis spectrophotometry at 255–260 nm. Removal efficiency was calculated and compared to synthetic solutions to assess the composite beads’ real-world performance.

### 2.6 Data Analysis

Adsorption isotherms were analyzed using both Langmuir and Freundlich models:

$$\mathrm{Langmuir}:q_{e}=\frac{q_{max}K_{L}C_{e}}{1+K_{L}C_{e}}$$

$$\mathrm{Freundlich}:q_{e}=K_{F}C_{e}^{1/n}$$

where *q*_*e*_ (mg·g^–1^) is the equilibrium adsorption capacity, *q*_max_ is the maximum monolayer capacity, *K*_*L*_ (L/mg) is the Langmuir constant, *K*_*F*_ is the Freundlich constant, and 1/*n* represents adsorption intensity.

Kinetic data were fitted to both pseudo-first-order and pseudo-second-order models:

$$\mathrm{Pseudo‐first‐order}:\ln\left(q_{e}-q_{t}\right)=\ln q_{e}-k_{1}t$$

$$\mathrm{Pseudo‐second‐order}:\frac{t}{q_{t}}=\frac{1}{k_{2}q_{e}^{2}}+\frac{t}{q_{e}}$$

where *q*_*t*_ (mg·g^–1^) is the adsorption capacity at time *t, k*_1_ (min^–1^) is the first-order rate constant, and *k*_2_ (g·mg^–1^·min^–1^) is the second-order rate constant.

All experiments were conducted in triplicate, and mean values with standard deviations are reported. Nonlinear regression was performed using OriginPro 2025. The correlation coefficient (*R* ^2^) and error analysis (χ^2^ test, RMSE) were used to assess model accuracy.

## 3 Results and Discussion

### 3.1 Morphology and Structural Characterization

The synthesized gamma-chitosan/alginate composite beads, functionalized with kaolin and gallic acid (γCS-AG/KA-GA), exhibited a uniform spherical morphology with a smooth and compact surface. The prepared γCS-AG/KA-GA beads exhibited a uniform spherical shape, characterized by a smooth surface and a translucent appearance (see Figure 1). The average diameter of the hydrated beads ranged from 1.5 to 2.0 mm. The optical micrographs clearly illustrate a well-defined, round geometry and a dense internal structure, with no visible cracks or agglomeration. This indicates effective homogeneous cross-linking between alginate and γ-chitosan through Ca^2+^ coordination. The incorporation of kaolin clay enhanced the rigidity of the beads and reduced surface collapse. At the same time, gallic acid enhanced interactions between the polymer and inorganic components via hydrogen bonding and phenolic cross-linking. The compact inner texture observed in the magnified images suggests a robust composite network that can withstand deformation during handling and swelling in an aqueous solution. These morphological characteristics align with previous studies demonstrating that the inclusion of kaolin and polyphenolic agents enhances the structural integrity and homogeneity of biopolymer composites, leading to improved mechanical strength and enhanced adsorption stability.

**Figure 1:**
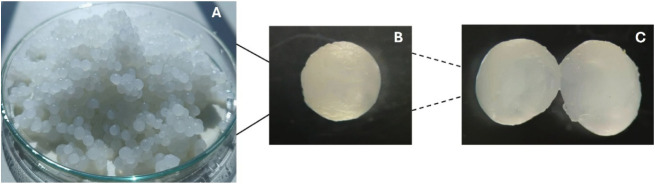
Morphology of γCS-AG/KA-GA composite beads: (A) overall view of hydrated beads showing a uniform spherical shape, (B) optical microscopy image of an individual bead, and (C) cross-sectional view revealing a dense internal structure.

HAADF-STEM, BEI-STEM, and HRTEM images of gCS-AG/KA-GA composite beads. Uniform kaolin dispersion and strong polymer-clay interfacial adhesion are observed. High-resolution HRTEM reveals lattice fringes corresponding to *d*(001) = 0.71 nm and *d*(002) = 0.36 nm of kaolinite, confirming the structural integrity of the clay phase within the γ-chitosan/alginate matrix. The HAADF-STEM image in the top left shows a densely packed bead surface with strong Z-contrast, indicating the uniform incorporation of kaolin clay platelets within the polymer matrix (Figure 2). The BEI-STEM image, positioned in the top middle, reveals a compact, wrinkle-free surface morphology with fine nodular features. This suggests homogeneous gelation and effective Ca^2+^ cross-linking between γ-chitosan and alginate. The HRTEM images, displayed in the top-right and bottom panels, exhibit an amorphous polymer background interspersed with darker layered domains corresponding to kaolin platelets. These lamellar regions are evenly distributed, with no visible aggregation, suggesting good compatibility and dispersion of the inorganic phase within the biopolymer network.

**Figure 2:**
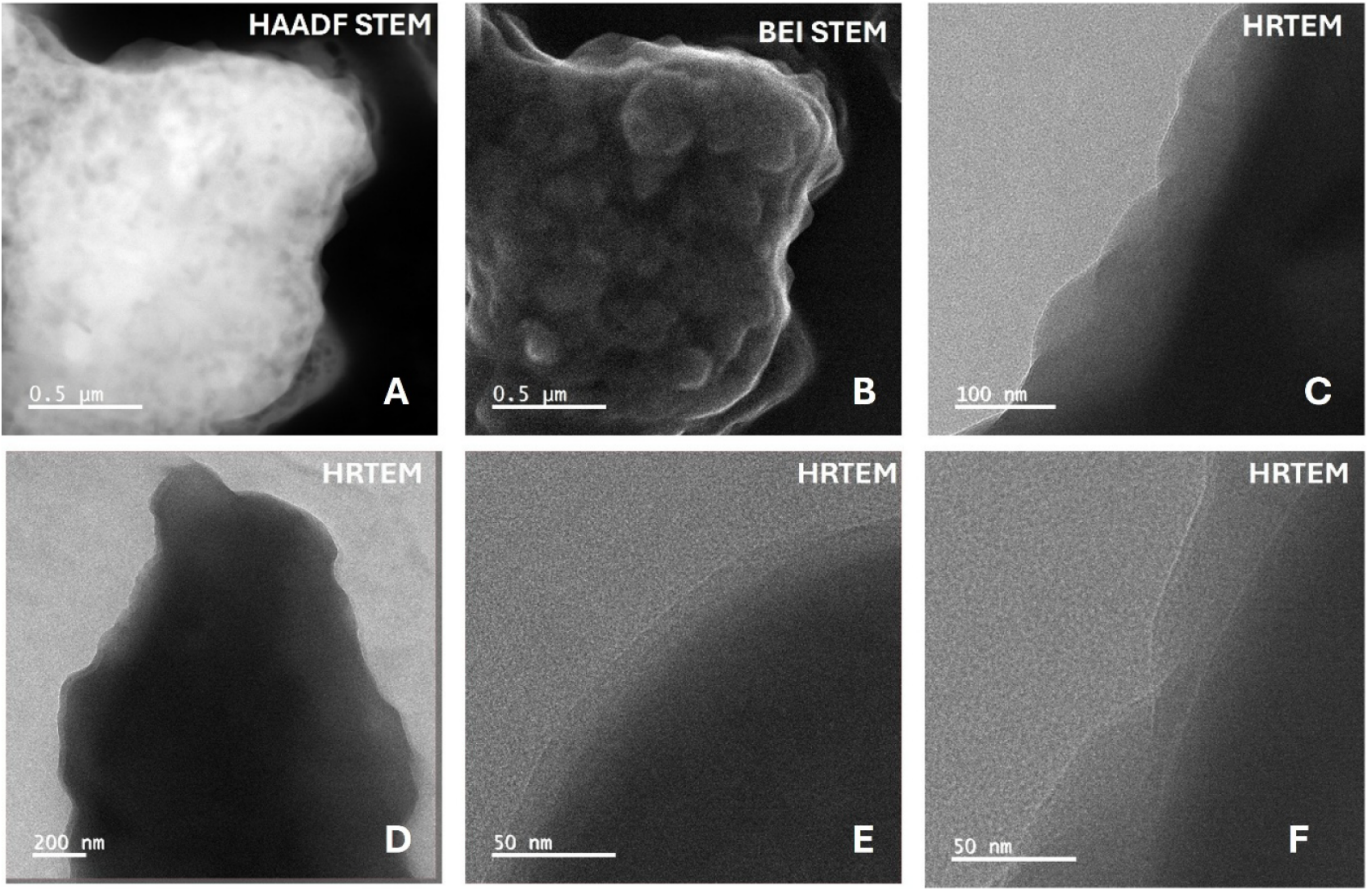
TEM and HRTEM characterization of the γCS-AG/KA-GA composite bead: (A) HAADF-STEM image showing the overall morphology and dense structure of the composite bead, (B) BEI-STEM image highlighting surface texture and compositional contrast, (C) low-magnification HRTEM image of the bead edge, (D) HRTEM image revealing the internal morphology, and (E, F) high-resolution HRTEM images showing the interfacial region and nanoscale structural features of the γCS-AG/KA-GA composite.

At higher magnification (shown in the bottom center and right), clear lattice fringes become visible, with *d*-spacing values of approximately 0.71 ± 0.03 nm and 0.36 ± 0.02 nm. These values correspond to the (001) and (002) planes of kaolinite, respectively (Figure 3). The continuous lattice fringes extending from the clay phase into the polymer matrix indicate strong interfacial adhesion, likely mediated by hydrogen bonding and π–π interactions involving the phenolic groups of gallic acid. These observations confirm that the kaolin-GA reinforcement creates a stable, nanostructured hybrid interface, enhancing both mechanical integrity and adsorption performance.

**Figure 3:**
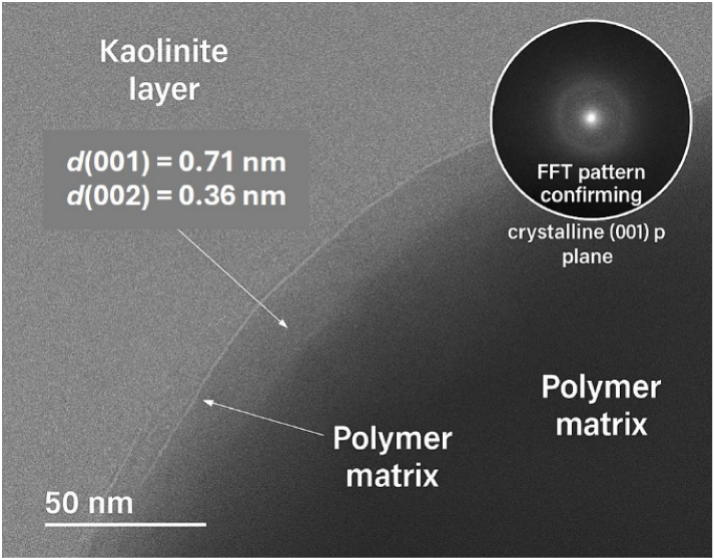
High-resolution TEM image of γCS-AG/KA-GA composite bead showing clear lattice fringes of kaolinite with *d*(001) = 0.71 nm and *d*(002) = 0.36 nm, and FFT inset confirming crystalline order at the interface.

The EDS spectrum displays prominent peaks corresponding to various elements: O (0.53 keV), Al (1.49 keV), Si (1.74 keV), Ca (3.69 keV), and C (0.28 keV). These peaks confirm the presence of both organic and inorganic components within the composite matrix (Figure 4). The detection of aluminum (Al) and silicon (Si) is attributed to kaolinite (Al_2_Si_2_O_5_(OH)_4_), while calcium (Ca) is derived from calcium cross-linking (Ca^2+^ ions) between alginate and γ-chitosan. The signals for carbon (C) and oxygen (O) represent the biopolymer backbone, which consists of chitosan, alginate, and gallic acid functional groups.^24^ Elemental mapping images (shown on the right) further validate the uniform distribution of Al, Si, Ca, and O across the bead surface. This indicates that the kaolin platelets are well-dispersed within the polymer matrix, rather than forming agglomerated clusters.^17,25^ The colocalization of Si and Al demonstrates that kaolin maintains its structural integrity after composite formation. Additionally, the overlap of the Ca and Si regions suggests a strong electrostatic association between Ca^2+^ ions and negatively charged sites on the kaolin surface. No significant impurities were detected, indicating the purity of the synthesized material. The mapping pattern supports the formation of a uniform hybrid network, in which kaolin provides structural reinforcement, and gallic acid enhances interfacial bonding between the polymer and clay via hydrogen bonding and chelation.

**Figure 4:**
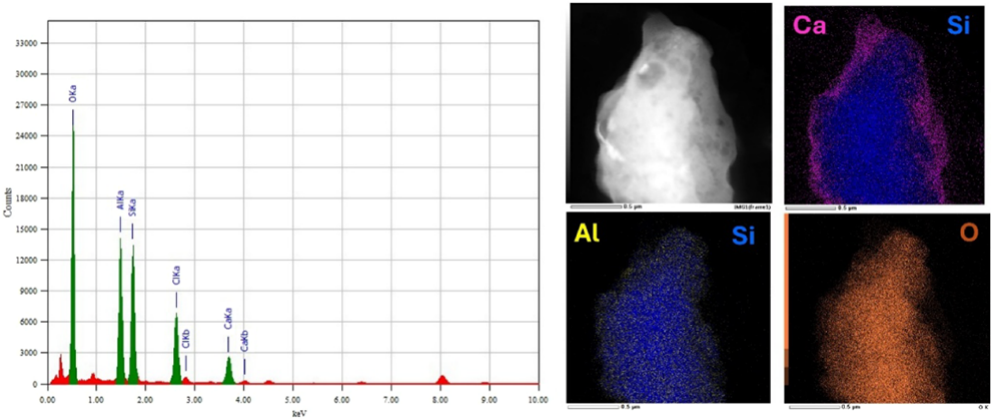
EDS spectrum and elemental mapping of γCS-AG/KA-GA composite beads.

BET analysis revealed an increase in surface area from 51.447 m^2^·g^–1^ (alginate control) to 71.405 m^2^·g^–1^ for γCS-AG/KA-GA, along with higher pore volume and slightly larger average pore diameter (Table 1). The textural enhancement is attributed to (i) kaolin acting as a rigid scaffold that prevents pore collapse and (ii) GA-driven secondary interactions that increase cross-link density while preserving accessible porosity.

**Table 1:** BET Surface Area and Pore Structure Comparison of Alginate, γCS-AG, and γCS-AG/KA-GA Composite Beads

Samples	Surface Area^a^, S_BET_ (m^2^·g^–1^)	Pore Volume^b^, Vp (cm^3^·g^–1^)	Average Pore diameter^b^, Dp (nm)
Alginate beads (control)	51.447	0.058	2.969
γCS-AG bead	68.405	0.083	2.971
γCS-AG/KA-GA beads	71.405	0.094	2.977

a Specific surface area (S_BET_) determined by the Brunauer–Emmett–Teller (BET) method using N_2_ adsorption–desorption isotherms.

b Total pore volume (Vp) and average pore diameter (Dp) calculated from the adsorption branch of the N_2_ isotherm using the Barrett–Joyner–Halenda (BJH) method.

As shown in Figure 5, the diffractogram of AG beads showed two broad peaks centered at 2θ ≈ 14°–24°, characteristic of the semiamorphous structure of calcium alginate. When γ-irradiated chitosan (γCS-AG beads) was incorporated, the intensity slightly increased near 2θ ≈ 20°. This change indicates partial ordering and hydrogen bonding alignment between the −NH_2_ groups of chitosan and the −COO^–^ groups of alginates. In contrast, the γCS-AG/KA-GA beads exhibited distinct sharp peaks at 2θ ≈ 20.1°, 25.0°, and 35.1°, which correspond to the crystalline planes (001), (002), and (110) of kaolinite (Al_2_Si_2_O_5_(OH)_4_).^17^ The reduced intensity of the polymer peaks, along with the broadening in this region, suggests that kaolin was successfully dispersed within the polymer matrix, forming an intercalated hybrid structure. Furthermore, the overall reduction in the amorphous background of γCS-AG/KA-GA indicates an enhancement in crystallinity due to the reinforcement from kaolin and cross-linking with gallic acid. This process tightens the packing of the polymer chains through hydrogen bonding and π–π stacking. These structural modifications result in enhanced mechanical strength and a reduced swelling ratio, as confirmed by both BET and mechanical data.

**Figure 5:**
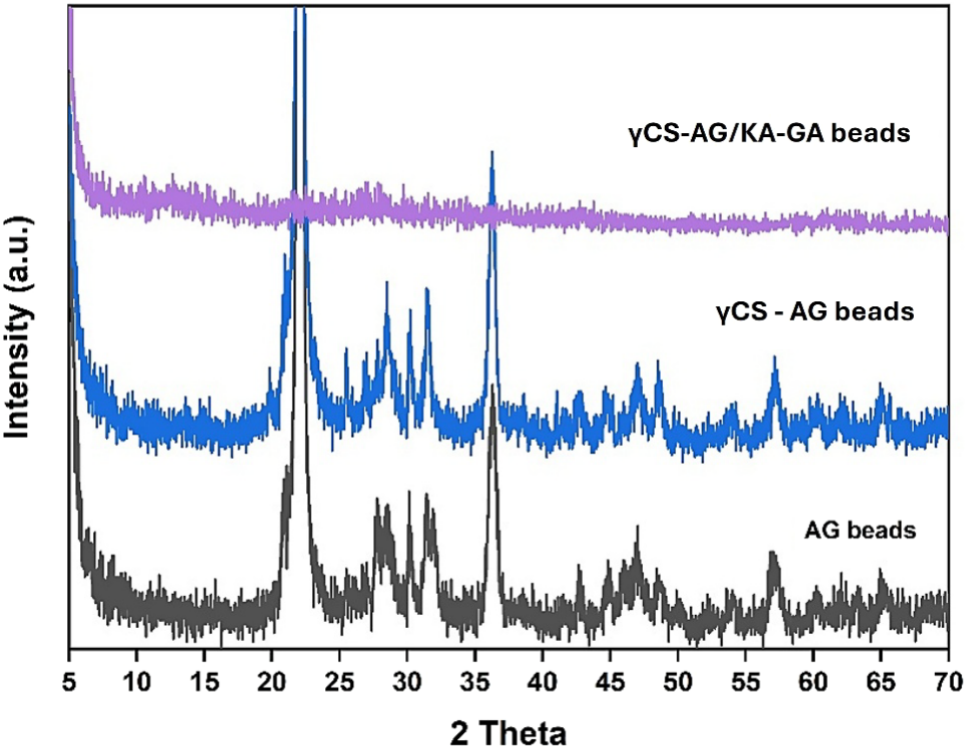
XRD patterns of alginate (AG), γ-chitosan/alginate (γCS-AG), and kaolin-gallic acid-reinforced γ-chitosan/alginate (γCS-AG/KA-GA) composite beads.

The FTIR spectra illustrate (Figure 6) the structural changes and successful incorporation of both inorganic and phenolic modifiers within the biopolymer network. In γ-chitosan (γCS), characteristic bands were observed at approximately 3420 cm^–1^ (O–H/N–H stretching), 1655 cm^–1^ (amide I, C=O stretching), and 1595 cm^–1^ (N–H bending of amide II). For alginate (AG) beads, intense absorption peaks were noted at 1625 cm^–1^ and 1415 cm^–1^, which correspond to the asymmetric and symmetric stretching vibrations of carboxylate groups (−COO^–^) in Ca-alginate. This confirms the ionic cross-linking with Ca^2+^. When γ-chitosan was combined with alginate (γCS-AG beads), the broad O–H/N–H band shifted slightly to around 3385 cm^–1^, and the carboxylate bands were enhanced. This indicates the formation of electrostatic and hydrogen bonding interactions between the −NH_3_^+^ groups (from chitosan) and the −COO^–^ groups (from alginate). The γCS-AG/KA-GA beads displayed additional distinct features. The O–H/N–H stretching peak became broader (centered at approximately 3395 cm^–1^) due to extensive hydrogen bonding between the polymeric hydroxyls, kaolin surface −OH groups, and phenolic groups of gallic acid.

**Figure 6:**
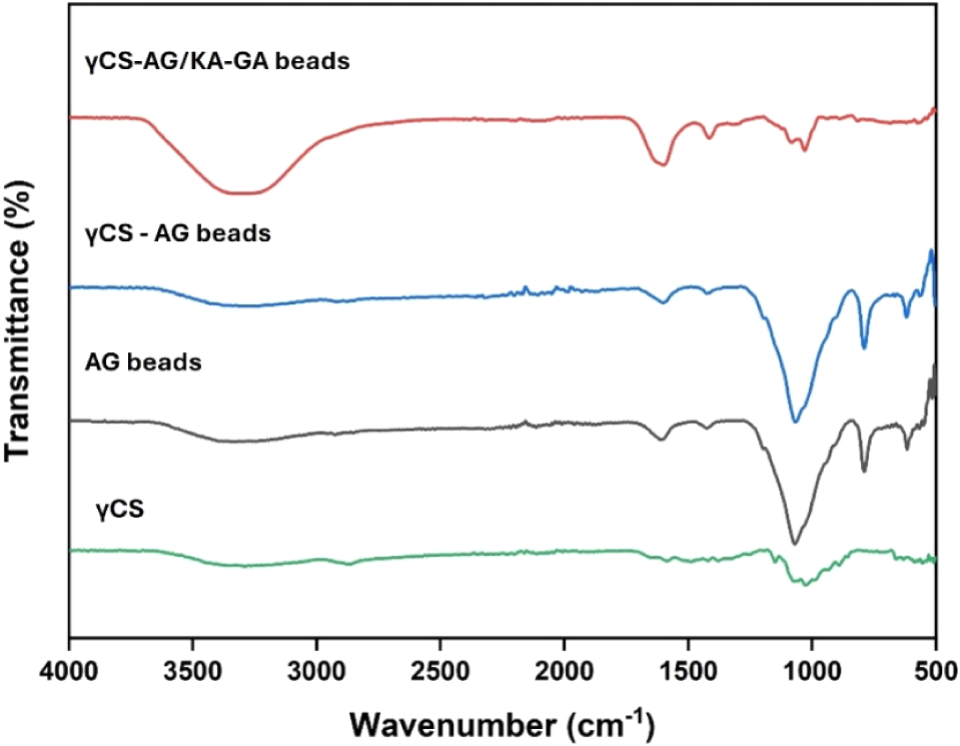
FTIR spectra of γ-chitosan (γCS), alginate (AG), γCS-AG, and kaolin/gallic acid-reinforced composite beads (γCS-AG/KA-GA).

Furthermore, a new shoulder near 1245 cm^–1^ was observed, corresponding to the C–O stretching of phenolic ester/phenolate, which confirms the incorporation of gallic acid. The band in the range of 1020–1035 cm^–1^ intensified, associated with Si–O–Si stretching from kaolin, supporting the successful embedding of inorganic components.^17^ These spectral changes confirm chemical interactions and cross-linking among γ-chitosan, alginate, kaolin, and gallic acid, resulting in a denser, more organized polymer network.

### 3.2 Adsorption of Chlorhexidine and Cetylpyridinium

The UV–vis analytical method used to determine the concentrations of CHG and CPC showed good linearity within the tested concentration range. The method demonstrated adequate sensitivity, with acceptable limits for detection and quantification. Recovery experiments conducted using spiked dental wastewater matrices confirmed satisfactory analytical accuracy, indicating that the UV–vis method is suitable for monitoring CHG and CPC concentrations in complex wastewater samples. The adsorption kinetics of CHG and CPC showed rapid uptake within the first 60 min, followed by gradual attainment of equilibrium at approximately 180 min (see Figure 7a). The initial fast phase is attributed to the abundant accessible active sites on the bead surface. In contrast, the subsequent slower phase suggests that diffusion processes play a significant role as equilibrium is approached. This behavior aligns with the kinetic modeling results, which showed that the pseudo-second-order model provided the best fit. In addition, intraparticle diffusion analysis indicates that the adsorption process occurs in multiple steps rather than a single diffusion-controlled mechanism. The equilibrium adsorption capacities, as determined by Langmuir isotherm analysis, were 82.7 mg·g^–1^ for CHG and 75.3 mg·g^–1^ for CPC. These values indicate a strong affinity between the cationic antiseptics and the functionalized composite beads, reflecting favorable monolayer adsorption on energetically uniform sites, which is supported by the good agreement between the experimental data and the Langmuir model. The pH-dependent adsorption behavior (Figure 7b) revealed that maximum adsorption occurred at near-neutral pH (6–7). This trend is consistent with the pH-dependent ionization of surface functional groups (−COO^–^ from alginate and −NH_2_/–NH_3_^+^ from chitosan), as well as the reported surface charge behavior of alginate-chitosan systems and kaolin-containing composites. At acidic pH, protonation of surface functional groups and increased competition with H^+^ ions reduce the effective electrostatic attraction toward the cationic CHG and CPC molecules. Conversely, at alkaline conditions (pH > 8), alterations in surface charge distribution weaken the ionic interactions, resulting in decreased adsorption efficiency. Therefore, near-neutral conditions strike an optimal balance for both electrostatic attraction and hydrogen-bonding interactions. Increasing ionic strength from 0 to 100 mM NaCl resulted in a moderate decrease in removal efficiency, approximately 10–15%, as illustrated in Figure 7c. This reduction can be attributed to charge-screening effects and to competition between Na^+^ ions and cationic antiseptics for available adsorption sites. This observation highlights the significant role of electrostatic interactions in the adsorption process and helps explain the decreased removal efficiency observed in real dental wastewater, which contains various inorganic ions and organic components. Overall, electrostatic attraction is identified as the primary mechanism for adsorption, while hydrogen bonding and π–π interactions associated with gallic acid functional groups provide additional stabilization.

**Figure 7:**
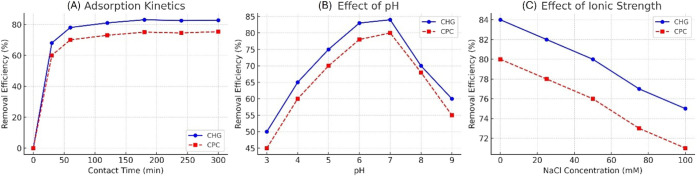
Adsorption kinetics and pH-ionic strength effects of CHG and CPC on γCS-AG/KA-GA composite beads: (A) adsorption kinetics, (B) effect of pH, and (C) effect of ionic strength on CHG and CPC removal. Experimental conditions: 25 mL of 1 mg·L^–1^ CHG or CPC, adsorbent dosage of 3.0 g, agitation speed of 250 rpm, and temperature of 25 ± 1 °C.

The reusability of the γCS-AG/KA-GA composite beads was assessed through consecutive adsorption–desorption cycles under the same operating conditions. The removal efficiency decreased from approximately 85% in the first cycle to 71% in the second and 59% in the third. After the fourth cycle, we observed noticeable deformation and a loss of structural integrity in the beads, indicating mechanical degradation. The gradual decline in adsorption performance can be attributed to the partial saturation of active sites and mechanical stress incurred during repeated regeneration. These results show that the composite beads maintain a reasonable adsorption capacity for up to 3 reuse cycles, suggesting potential for practical application. However, further improvements in mechanical stability would be necessary for extended reuse.

The contribution of gallic acid is attributed to its multiple phenolic groups, which provide more effective hydrogen bonding and π–π interactions than less functionalized phenolic modifiers reported in previous biopolymer-based adsorbents. Postadsorption FTIR and EDS analyses indicate the involvement of −COO^–^ and −NH functional groups in CHG/CPC binding, as well as the appearance of Cl signals after adsorption, confirming successful immobilization of the antiseptics on the composite surface.

Characterization after adsorption revealed significant structural and compositional changes in the γCS-AG/KA-GA beads. HRTEM and STEM images revealed smoother surfaces with a thin coating layer resulting from the adsorption of CHG and CPC molecules (Figure 8). EDS confirmed the presence of chlorine (Cl), which was not detectable prior to adsorption, indicating the successful immobilization of both CHG and CPC, as they contain Cl in their molecular structures. The XRD pattern showed reduced peak intensities and increased amorphous content, suggesting that the incorporation of CHG and CPC molecules disrupted the structure. In FTIR spectra, new C–H and C–N bands appeared. At the same time, the intensity of −COO^–^ and −NH peaks decreased, confirming the presence of electrostatic and hydrogen-bonding interactions between the antiseptics and the polymeric matrix.

**Figure 8:**
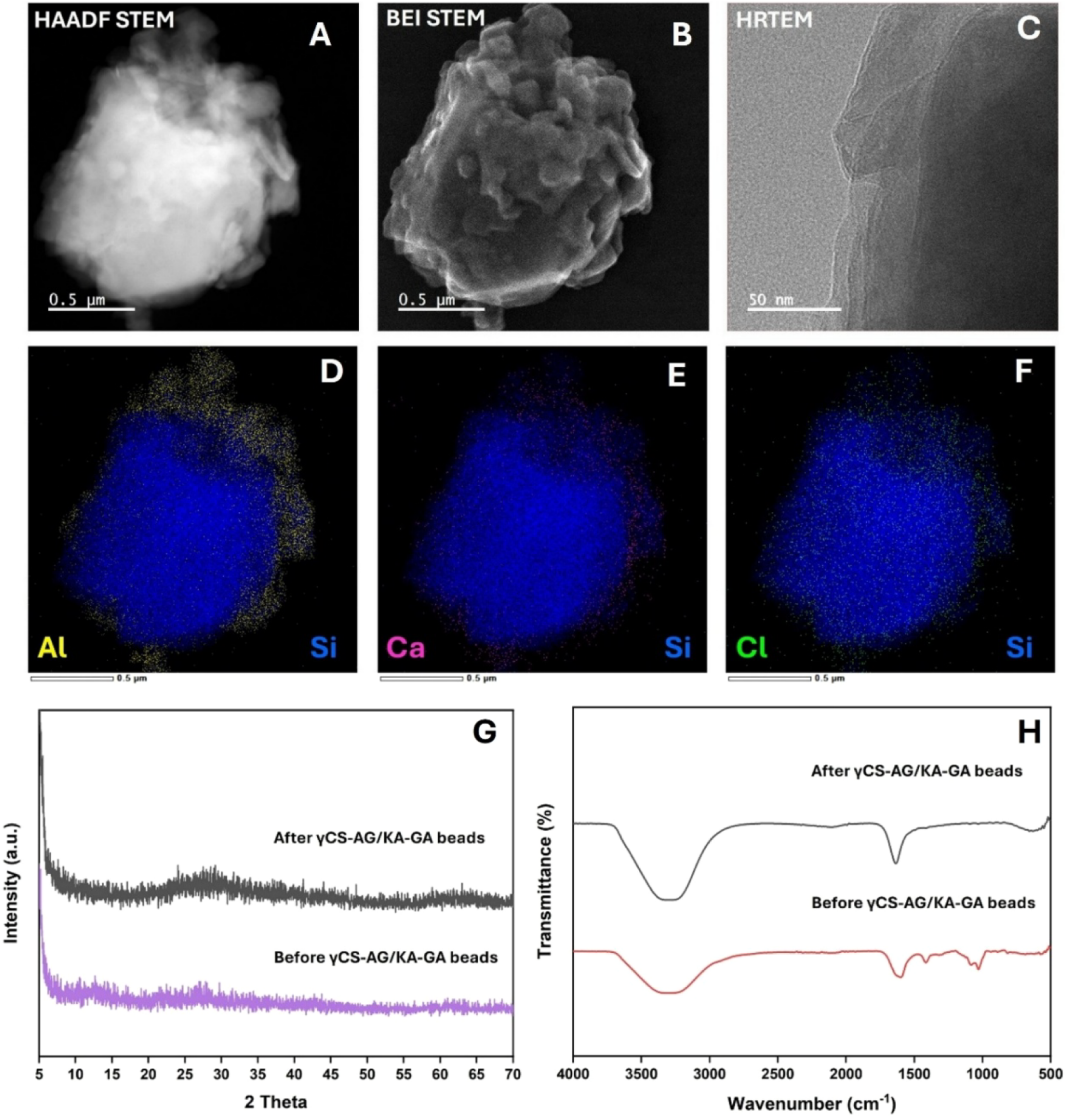
Postadsorption characterization of γCS-AG/KA-GA composite beads after CHG and CPC uptake: (A) HAADF-STEM image showing the overall morphology of the composite bead, (B) BEI-STEM image highlighting surface contrast and texture, (C) HRTEM image of the bead edge, (D) Al/Si elemental mapping, (E) Ca/Si elemental mapping, and (F) Cl/Si elemental mapping confirming the immobilization of CHG/CPC molecules. (G) XRD patterns before and after adsorption indicate an increase in amorphous character, and (H) FTIR spectra before and after adsorption show changes in the −COO^–^ and −NH bands associated with electrostatic interactions and hydrogen bonding.

### 3.3 Adsorption Isotherms and Kinetics

The equilibrium data were well fit by the Langmuir model (*R* ^2^ > 0.98), indicating that monolayer adsorption occurs on uniform active sites (Figure 9). The maximum adsorption capacities (*q*_max_) were found to be 82.7 mg·g^–1^ for CHG and 75.3 mg·g^–1^ for CPC. The *q*_*e*_ versus *C_e_* relationship was evaluated through the linearized Langmuir representation (*C_e_*/*q*_*e*_ versus *C_e_*), which was employed to calculate the Langmuir parameters, including *q*_max_. A literature comparison (Table 2) has been added to benchmark the adsorption capacity of γCS-AG/KA-GA against previously reported adsorbents for CHG/CHX and CPC-related cationic compounds. While variations in experimental conditions, such as initial concentration, dosage, and matrix composition, can influence direct comparisons, the *q*_max_ values obtained in this study are within the upper range of reported performances. The adsorption capacities obtained from isotherm analysis are consistent with postadsorption FTIR and EDS results, which confirm the involvement of surface functional groups and the presence of CHG/CPC-related elements on the composite beads. Furthermore, real wastewater tests support its applicability in competitive ionic and organic environments.

**Figure 9:**
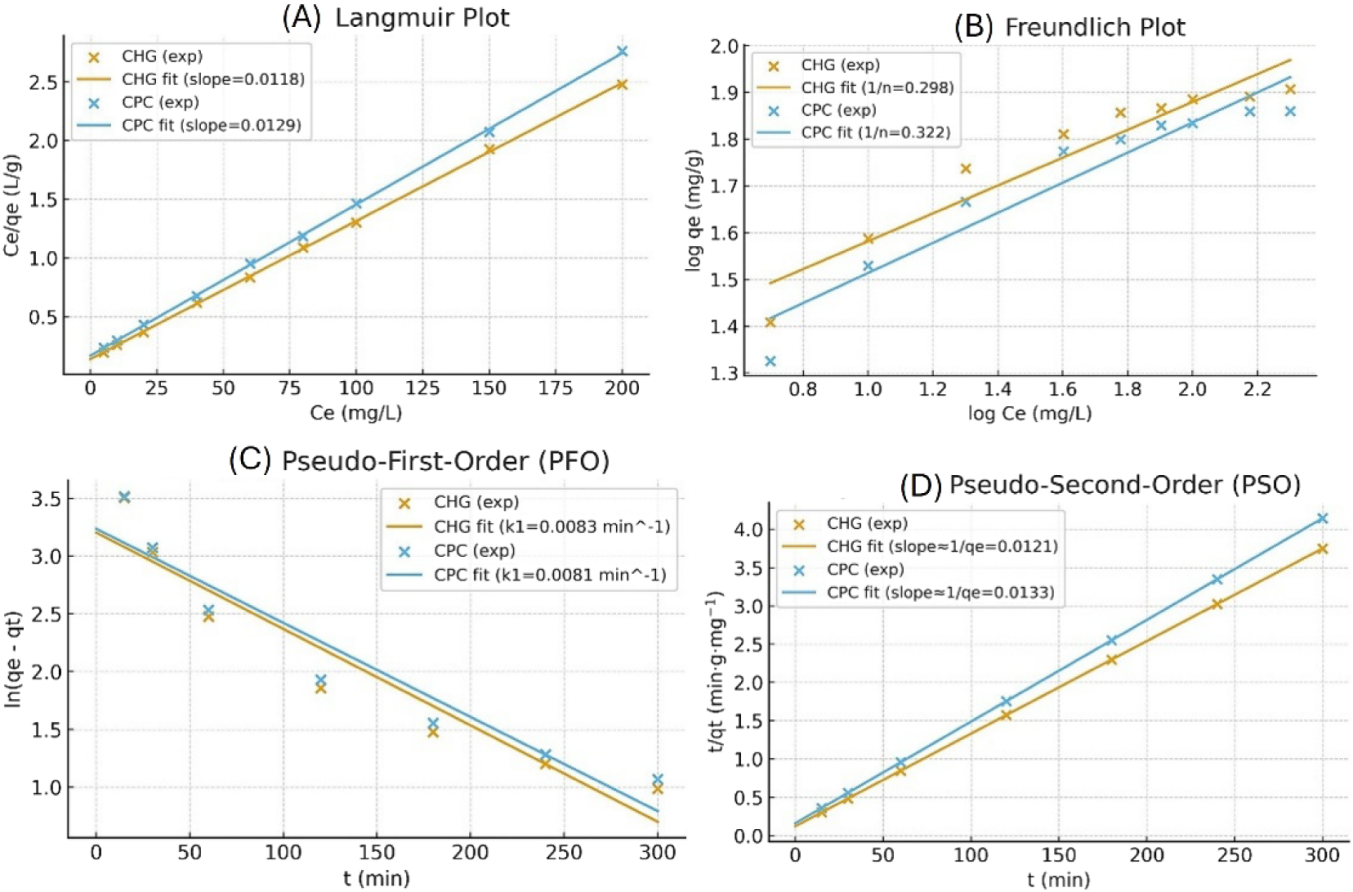
Adsorption isotherms and kinetic models of CHG and CPC. (A) Linearized Langmuir isotherm (*C_e_*/*q*_*e*_ versus *C_e_*), (B) Freundlich isotherm, (C) Pseudo-first-order kinetic model, and (D) Pseudo-second-order kinetic model. Experimental conditions: 25 mL of 1 mg·L^–1^ CHG/CPC, adsorbent dosage of 3 g, temperature 25 ± 1 °C, pH 7, and agitation speed of 250 rpm.

**Table 2:** Comparison of Reported Adsorbents and This Work^a^

Target	Adsorbent (reported)	Model/Key finding	*q*_max_ (mg·g^–1^)	Ref
CHD	Chitosan-magnetic iron oxide nanoparticles	Computational simulations	73.86	^26^
CHG/CHX	Nitrogen-doped chitin (from shells)	Langmuir + PSO; postadsorption EDX shows N/Cl	94.92	^27^
CHX/OCT	Heterogeneous biodegradation by fungi	Dental care antimicrobial agents chlorhexidine	38.92	^1^
CHG	γCS-AG/KA-GA beads (this work)	Langmuir + PSO (best fits)	82.7	(this work)
CPC	γCS-AG/KA-GA beads (this work)	Langmuir + PSO (best fits)	75.3	(this work)

a Direct comparison should be made with caution due to differences in experimental conditions (initial concentration, adsorbent dosage, and matrix composition).

As summarized in Table 2, the γCS-AG/KA-GA composite beads exhibit adsorption capacities for CHG and CPC that are comparable to or exceed those of several previously reported adsorbents. Although some materials show higher *q*_max_ values under optimized laboratory conditions, the present system demonstrates competitive performance while maintaining structural simplicity and applicability to real dental wastewater matrices.

The adsorption capacities stem from the increased surface area and the synergistic effects of kaolin and gallic acid functionalization, which enhance the interaction between the composite surface and cationic CHG/CPC molecules. Kinetic modeling revealed that the pseudo-second-order (PSO) model (*R* ^2^ ≈ 0.99) best fit the experimental data, suggesting that surface interactions, such as electrostatic attraction and hydrogen bonding, play a dominant role in the adsorption process.^28^ To further examine the adsorption mechanism, the kinetic data were also evaluated using intraparticle diffusion and Elovich models. The intraparticle diffusion plots did not pass through the origin, indicating that adsorption proceeds via a multistep mechanism rather than a single diffusion-controlled process.

The linearized Langmuir plot was used to determine the maximum adsorption capacity (*q*_max_, mg·g^–1^). At the same time, the Langmuir and PSO models provided the best fits, confirming monolayer adsorption dominated by electrostatic and hydrogen-bonding interactions.

### 3.4 Application to Real Dental Wastewater

Under the same conditions used for synthetic tests (3.0 g of beads per 25 mL, 25 °C, 250 rpm, for 180 min), the removal efficiency in actual effluents collected from the BTU Dental Clinic reached 65 ± 3%. This performance was lower than that observed in synthetic solutions, which achieved approximately 83% removal for CHG and around 75% for CPC. The decreased efficiency can be attributed to several factors: 1) Matrix effects and competitive adsorption due to dissolved salts and organic matter. 2) Micelle formation by surfactants, which can trap CHG and CPC, hindering pore diffusion. 3) Suboptimal pH and elevated ionic strength, leading to charge screening. 4) Colloidal fouling that blocks active sites and affects UV–vis readings. 5) Insufficient residence time or adsorbent dosage in a complex matrix.

For future work, several process intensification options are suggested: adjusting the pH to between 6.5 and 7.0, increasing the adsorbent dosage to 4–5 g per 50 mL, extending the contact time to 240–300 min, prefiltration with low-dose activated carbon to reduce surfactants, prerinsing the beads to mitigate fouling, and implementing a two-stage batch configuration.

### 3.5 Proposed Adsorption Mechanism

The proposed adsorption mechanism is illustrated in Figure 10. The adsorption mechanism involves several synergistic interactions. First, there is electrostatic attraction between the negatively charged −COO^–^ groups of alginate and the cationic ammonium groups of CHG and CPC. Additionally, hydrogen bonding occurs between the hydroxyl and amine groups of γ-chitosan/gallic acid and the functional groups of the antiseptic molecules.

**Figure 10:**
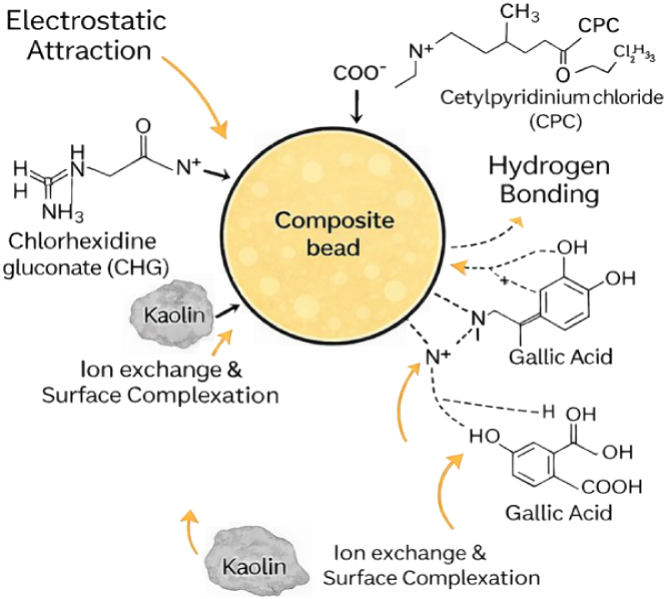
Proposed adsorption mechanism of CHG and CPC onto γCS-AG/KA-GA composite beads.

Furthermore, π–π interactions take place between the aromatic ring of gallic acid and the aromatic/pyridinium moieties of CHG and CPC. The aluminosilicate sites in kaolin also enhance the process by facilitating ion exchange and surface complexation. Overall, these interactions significantly improve the adsorption capacity and structural stability of the γCS-AG/KA-GA composite beads compared to traditional chitosan- or alginate-based systems.

## 4 Conclusion

In this study, we successfully synthesized kaolin-gallic acid functionalized gamma-irradiated chitosan/alginate composite beads (γCS-AG/KA-GA) and demonstrated their effectiveness as an eco-friendly adsorbent for removing cationic dental antiseptics from aqueous systems, including real dental wastewater. Comprehensive structural and physicochemical characterizations confirmed the formation of a stable hybrid biopolymer network, with kaolin and gallic acid homogeneously distributed throughout. This distribution contributed to enhanced structural integrity and improved adsorption functionality. The adsorption process was driven by synergistic mechanisms, including electrostatic attraction, hydrogen bonding, and π–π interactions among the functional groups of alginate, gamma-irradiated chitosan, kaolin, and gallic acid. The composite beads showed reliable performance in complex wastewater conditions, highlighting their robustness and practical applicability. Overall, these findings underscore the potential of this biopolymer-based composite as a sustainable, low-cost, and environmentally friendly material for addressing persistent antiseptic contamination in dental and healthcare wastewater management systems.

## Data Availability

Data are available upon request from the authors.
